# Incidence and impact on prognosis of peri-procedural myocardial infarction in 2760 elective patients with stable angina pectoris in a historical prospective follow-up study

**DOI:** 10.1186/s12872-016-0293-4

**Published:** 2016-06-16

**Authors:** Martin Kirk Christensen, Hui Huang, Christian Torp-Pedersen, Torleif Trydal, Jan Ravkilde

**Affiliations:** Department of Cardiology, Aalborg University Hospital, Aalborg, Denmark; General Hospital of Ningxia Medical University, Yinchuan, China and Visiting Doctor at Department of Cardiology, Aalborg University Hospital, Aalborg, Denmark; Department of Health Science and Technology, Aalborg University, Aalborg, Denmark; Department of Clinical Biochemistry, Aalborg University Hospital, Aalborg, Denmark

**Keywords:** Troponin, Elective, Prognosis, Myocardial infarction, Peri-procedural, Percutaneous coronary intervention

## Abstract

**Background:**

The clinical significance of myocardial infarction related to treatment with percutaneous coronary intervention (PCI) has been subject of great discussion. This subject has been studied for many years using different definitions of peri-procedural myocardial infarction and different biomarkers, the results have varied greatly depending on methods and time of the study. This study was to determine the incidence and prognostic significance of elevated cardiac biomarkers after elective PCI in patients with stable angina pectoris using the current cut-off set by the Third Universal Definition of Myocardial Infarction and current biomarkers.

**Methods:**

We performed a historical prospective follow-up study of all patients with stable angina pectoris who underwent elective PCI at Aalborg University Hospital, Denmark from January 1^st^ 2000 to December 31^st^ 2012. We stratified patients according to peak post-PCI troponin T (cTnT) and Creatine Kinase MB mass (CK-MBmass).

**Results:**

Follow-up for time to all-cause mortality was mean 5.8 years and total 15,891 years and mean 3.7 years and total 10,160 years for the combined endpoint of all-cause mortality and new onset heart failure. During the follow up period 399 of 2760 patients died (14.5 %) and 1095 (39.7 %) suffered the combined endpoint. Post-PCI concentration of cTnT and CK-MBmass was elevated above the defined cut-off in 419 patients (15.2 %) and 113 patients (4.1 %) respectively. There was no statistically significant difference between the groups in stratified analysis of the hazard rates by time regarding all-cause mortality for cTnT nor CK-MBmass. Regarding the combined endpoint the results were ambiguous. The results were unchanged in multivariable analyses that included age and gender.

**Conclusion:**

The incidence of elevated biomarkers after elective PCI in patients with stable angina pectoris using the defined cut-off (>5 x URL) was 15.2 % using cTnT and 4.1 % using CK-MBmass. The independent prognostic value for both cardiac biomarkers of any cut-off showed no statistical significance for all-cause mortality, whereas the combined endpoint (all-cause mortality or new-onset heart failure) were ambiguous in both short- and long-term follow-up.

## Background

Percutaneous coronary intervention (PCI) is an established procedure for the treatment of both stable angina pectoris and acute coronary syndrome, but the procedure is not without risk. An important complication to PCI is myocardial infarction (MI). This form of MI has been defined as MI type 4a by the Third Universal Definition of Myocardial Infarction Task Force Group [1]. Despite the clear definition, it remains unclear whether the chosen cut-off for biomarker concentration post-PCI is clinically and prognostically meaningful and should lead to changes in the treatment strategy [2].

The biomarker historically used for this purpose has been creatine kinase isoenzyme MB (CK-MB) initially as a measure of activity followed by analysis of mass concentration. Previous studies have shown a proportional relationship between rise in CK-MB and 6-month all-cause mortality [3, 4]. CK-MB has been largely replaced by high sensitive cardiac troponins T or I (cTn) as the biomarkers of choice in all types of myocardial infarction due to both higher sensitivity and specificity for myocardial necrosis [1, 2].

The aims of this study were to determine the incidence of elevated biomarkers post-PCI using the cut-off set by the Third Universal Definition of Myocardial Infarction in a cohort of patients with stable angina pectoris undergoing elective PCI and to determine the prognostic significance of this arbitrarily set limit of cardiac biomarker elevation defining peri-procedural MI by evaluating primarily all-cause mortality and secondarily a combined endpoint of all-cause mortality and heart failure.

## Methods

### Study design and inclusion

**Fig. 1:**
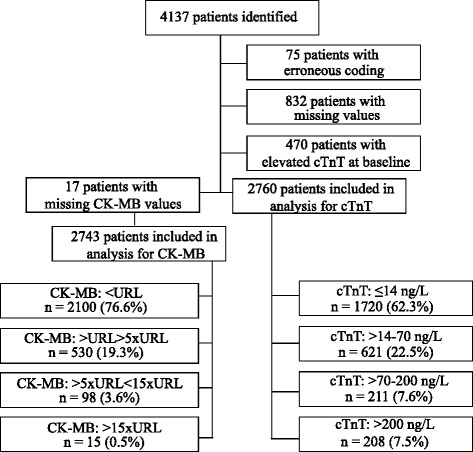
Study flow chart. cTnT = peak post procedural concentration of troponin T. CK-MB = peak post procedural concentration of CK-MBmass. URL = Upper reference limit

We performed a historical prospective follow up study of a cohort of patients with stable angina pectoris who underwent elective PCI at Aalborg University Hospital, Denmark from January 1^st^ 2000 to December 31^st^ 2012. Patients were eligible if the procedure was elective and there was no elevation of troponin prior to the procedure. Selection of the included 2760 patients can be followed in Fig. 1. As per department routine, patients had cTnT and CK-MBmass measured immediately before or during the procedure and 3–24 h later. In patients with more than one sample post-PCI, the highest value for cTnT was included. Patients who underwent several separate procedures were only included in analysis for the first intervention. All patients with a previous diagnosis of heart failure were excluded from analysis of the secondary endpoint of new-onset heart failure. As older studies have used CK-MB and not cTn, we also analyzed peak post-PCI CK-MBmass from the same cohort included in analysis for cTnT sampled from 3–24 h after the procedure.

### Biochemical analysis

cTnT and CK-MBmass were measured using a chemiluminiscence assay on either Elecsys 2010, Cobas 6000 or Cobas 8000 (Roche, Mannheim, Germany). The cTnT analysis changed to the new high sensitive version (hsTnT) in June 2010. Alignment of the concentrations between the cTnT and the hsTnT measurements was done according to a comparison study and information from the provider [5]. The 99^th^ percentile upper reference limit (URL) of hsTnT has been determined to be 14 ng/L, and the 99^th^ percentile of 4^th^ generation TnT analysis has been determined to less than 10 ng/L [5, 6].

Values up to 14 ng/l in hsTnT methods, or less than 10 ng/L in non-hsTnT methods, were defined as normal values. We considered that different versions of the TnT-assays had the same standardization from cTnT-values 70 ng/L and above. The cTnT value of 70 ng/L is also 5 x URL set by the Third Universal Definition Myocardial Infarction [1].

The CK-MB was measured as massconcentration and the assays underwent no change of standardization in the period in question. URL for CK-MBmass is gender specific and the current URL of 4 μg/L and 7 μg/L for woman and men (information from the provider, [6]), respectively, were used.

The analyses were performed as instructed by the provider. Internal quality controls at low and high concentrations, and external quality assessment (Labquality, Helsinki, Finland) were performed regularly throughout the period. The results were obtained from the clinical laboratory information system LABKA1 and LABKA2 databases [7].

### Follow-up

In this study data from one center, Aalborg University Hospital, was included. All patients were continuously registered in the Western Denmark Heart Registry immediately after the procedure [8]. In this registry patient data are available including risk factors, previous cardiac disease, clinical data e.g., body weight, blood pressure, latest P-Creatinine, indication, priority and procedural details such as number of treated vessels and details regarding type and number of stents used. Using the Western Denmark Heart Registry we were able to restrict our cohort to PCI performed on the indication stable angina pectoris that were performed electively.

To link databases and follow the patients we extracted information from the Danish National Civil Registration System, which identifies all Danish citizens with a unique personal identification number. Death is registered in this system within 2 weeks of death. The data, including date of birth, sex and date of death is updated continuously and is considered highly valid [9]. New onset heart failure required that the patient did not have a clinical diagnosis of heart failure prior to the procedure and further did use furosemide for a period of 60 days prior to the procedure. New onset heart failure was then considered to take place if the patient collected a prescription for furosemide or was admitted to hospital with a diagnosis of heart failure. Prescriptions were identified in the Danish Register of Medicinal Product Statistics, maintained by the Danish Medicines Agency. It contains information on all dispensed prescriptions from Danish pharmacies since 1994, including dosage, dispensing date and quantity of the drug dispensed. All drugs are categorized according to the Anatomical Therapeutic Chemical classification system [10].

Endpoints were chosen to all-cause mortality and the combined endpoint of all-cause mortality and new onset heart failure.

This study complies with the Declaration of Helsinki and STROBE guidelines. According to Danish law no specific approval from a local ethics committee or informed consent from the patients was required for this registry study. Permission was obtained to use data from Western Denmark Heart Registry. The study was approved by the Danish Data Protection Agency.

### Statistical analysis

Statistical analysis was performed in three and four groups after peak post-PCI concentrations of cTnT, the three groups were: ≤14 ng/L (URL), >14–70 ng/L and >70 ng/L (5x URL) and as four groups ≤14 ng/L (URL), >14–70 ng/L, >70–200 ng/L and >200 ng/L and four groups for CK-MBmass: < 4 μg/L (women) or 7 μg/L (men)), 4–20 μg/L or 7–35 μg/L; 20–60 μg/L or 35–105 μg/L and >60 μg/L or >105 μg/L for women and men, respectively. The values for CK-MBmass correspond to URL, 5 x URL and 15 x URL.

Comparison of baseline demographics was performed using rank-sum tests for continuous variables and chi-square for discrete variables. Survival curves were constructed from Kaplan Meier survival estimates and differences were analyzed using Cox Proportional Hazard Models. Analyses were performed with SAS (Statistical Analysis System, version 9.4, The SAS Institute) and R version 3.02 (R core development team) [11].

## Results

From January 1^st^ 2000 to December 31^st^ 2012; 4137 elective PCIs were performed on the diagnosis of stable angina pectoris, from this we identified 2760 patients eligible for further analysis (Fig. 1). Seventeen additional patients were excluded from analysis for CK-MBmass due to missing values. Follow-up for time to death was mean 5.8 years and total 15,891 years and mean 3.7 years and total 10,160 years for the combined endpoint of all-cause mortality and new onset heart failure. During the follow up period 399 patients died (14.5 %) and 1095 (39.7 %) suffered the combined endpoint. The incidence of elevated biomarkers post-PCI with cTnT exceeding URL was 1040/2760 (37.7 %) and 419/2760 (15.2 %) exceeding 5 x URL. Correspondingly for CK-MBmass 643/2743 (23.4 %) above URL and only 113/2743 (4.1 %) exceeded 5 x URL.

Table 1 shows the key elements of previous medical history, clinical and angiographic data in the four groups according to peak post-PCI cTnT. Of the 2760 included patients there was a high prevalence of risk factors for ischemic heart disease with male sex (71 %), age (mean 65 years), current smoking (25.9 %), diabetes (15.3 %), medically treated hypertension (64.3 %), medically treated hypercholesterolemia (70.8 %) and family history of coronary artery disease (49.2 %). The risk factors were evenly distributed among the groups with few statistically significant differences. The group with lowest cTnT were statistically significantly younger (64 years versus 67 years) than the other groups. Kidney function evaluated by P-Creatinine was also slightly but statistically significantly lower in the group with lowest cTnT (83 μmol/L versus 86 μmol/L). For severity of symptoms, all patients were evaluated by Canadian Cardiovascular Society classification of angina pectoris (CCS) with an even distribution and 87.7 % with angina pectoris CCS 1-2. As expected, the patients often had previous MI (26.6 %) and previous revascularization with PCI (23.4 %). There were statistically significantly more previous MIs in the group with cTnT >14–70 ng/L. With the lowest mean age in the group with cTnT ≤14 ng/L and the highest number of stents being used in the two groups with cTnT above 70 ng/L. Post-PCI peak values for cTnT were mainly distributed below the defined cut-off, but of the 2760 patients 419 (15.2 %) had elevated cTnT above 5 x URL post-PCI.

**Table 1:** Patient characteristics

Post-PCI peak TnT (ng/L)	≤14 (*n* = 1720)	>14-70 (*n* = 621)	>70-200 (*n* = 211)	>200 (*n* = 208)	*P* Value
Clinical data					
Age - years (IQR)	64 (57, 71)	67 (60, 74)	67 (59, 74)	67 (59, 73)	<0.0001
Male sex - no. (%)	1220 (70.9)	435 (70.0)	161 (76.3)	154 (74.0)	0.27
Angina severity CCS 1	247 (18.8)	94 (18.9)	28 (17.9)	26 (16.4)	
Angina severity CCS 2	913 (69.4)	365 (73.4)	111 (71.2)	121 (76.1)	
Angina severity CCS 3	151 (11.5)	36 (7.2)	17 (10.9)	11 (6.9)	
Angina severity CCS 4	4 (0.3)	2 (0.4)	0 (0.0)	1 (0.6)	0.34
Body Mass Index - kg/m^2^ (IQR)	26.3 (23.4, 29.3)	26.6 (23.5, 30.0)	25.8 (23.1, 29.2)	27.2 (24.4, 30.5)	0.23
P-Creatinine - μmol/L median (IQR)	83 (73, 96)	84 (74, 99)	85 (74, 100)	86 (75, 101)	0.01
Risk factors					
Smoking - Current - no. (%)	454 (27.2)	132 (21.8)	44 (21.2)	55 (26.8)	0.22
Non-insulin dependent diabetes- no. (%)	177 (10.5)	54 (9.1)	21 (10.3)	20 (10.0)	
Insulin dependent diabetes - no. (%)	80 (4.8)	34 (5.7)	9 (4.4)	10 (4.9)	0.62
Medically treated hypertension - no. (%)	1038 (62.8)	399 (67.2)	133 (64.3)	134 (66.3)	0.19
Medically treated hypercholesterolemia - no. (%)	1166 (70.5)	428 (71.6)	145 (70.0)	138 (68.3)	0.92
Family history of coronary artery disease - no. (%)	822 (49.6)	289 (48.5)	91 (44.2)	94 (46.5)	0.13
Medical history					
Previous myocardial infarction - no. (%)	439 (25.5)	184 (29.6)	59 (28.0)	51 (24.5)	0.034
Previous percutaneus coronary intervention - no. (%)	397 (23.9)	146 (24.4)	54 (26.1)	48 (23.8)	0.93
Medically treated heart failure	135 (7.8)	66 (10.6)	21 (10.0)	26 (12.5)	0.11
Procedure					
Stents used: 0 - no. (%)	60 (5.2)	32 (6.2)	5 (3.1)	9 (5.4)	
Stents used: 1 - no. (%)	751 (64.7)	269 (52.1)	57 (35.8)	51 (30.7)	
Stents used: 2-3 - no. (%)	316 (27)	179 (34.7)	75 (47)	74 (45)	
Stents used: >3 - no. (%)	34 (2.9)	36 (7.0)	22 (14)	32 (19)	<0.0001

*IQR* Interquartile range, *CCS* Canadian Cardiovascular Society classification of angina pectoris. *P* values reflects chi-square statistics comparing the five groups

**Fig. 2:**
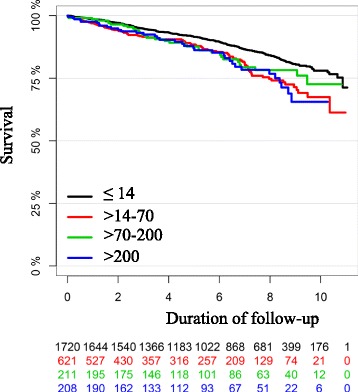
Kaplan-Meier curve of all-cause mortality by peak concentration of troponin T in ng/L. Each colored line representing one group according to peak concentration of troponin T. Duration of follow up in years from index procedure. Below the Kaplan-Meier curve is the number of patients still available for follow-up in each group

**Fig. 3:**
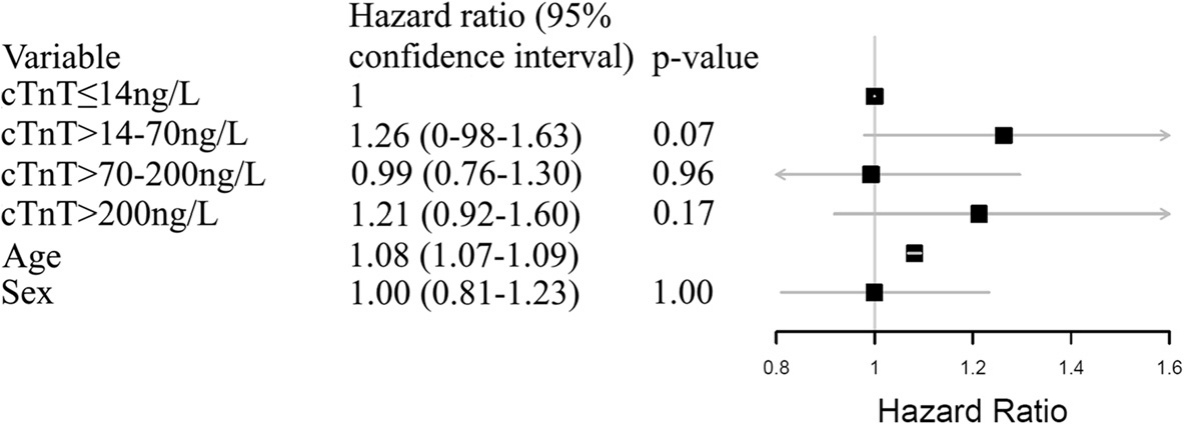
Forest Plot displaying Multivariate Cox Regression analysis for all-cause mortality. cTnT = peak post procedural concentration of troponin T in ng/L. Age signifies risk pr. year and sex risk associated with being male

**Fig. 4:**
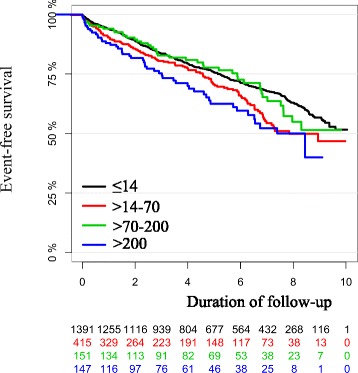
Kaplan-Meier curve of the combined endpoint of all-cause mortality or new onset heart failure by peak concentration of troponin T in ng/L. Each colored line representing one group according to peak concentration of troponin T. Duration of follow-up in years from index procedure. Below the Kaplan-Meier curve is shown the number of patients still available for follow-up in each group

The Kaplan-Meier curve of all-cause mortality by peak concentration of troponin T showed no sign of separation between the curves until after approximately 6 years (2000 days). After this time point, there was a slight tendency towards lower all-cause mortality in the group with cTnT ≤14 ng/L, but this was not statistically significant and there were relatively few patients available for follow up in each of the other three groups at this time (Fig. 2). The results were unchanged in multivariable analyses that included age and gender (Fig. 3). The corresponding curves for the combined endpoint of all-cause mortality and new onset heart failure showed early separation of the curves but with an unclear pattern. The group with post-PCI cTnT >70–200 ng/L was without significant separation from the group with cTnT ≤14 ng/L. However, the groups with cTnT >14–70 ng/L and above 200 ng/L did show separation of the curves throughout the follow-up period (Fig. 4).

In stratified analysis the hazard rates for time until death after post-PCI, peak cTnT was compared to the strata with cTnT < URL. There were no statistically significant differences regarding the primary endpoint of all-cause mortality with hazard ratios (HR) respectively in order of rising cTnT 1.62 (95 % confidence interval (CI) 0.97–1.63 *p* = 0.07), 0.99 (95 % CI 0.76–1.30 *p* = 0.96) and 1.21 (95 % CI 0.92–1.60 *p* = 0.17).

There were a few statistically significant differences when analyzing the combined endpoint of all-cause mortality or new onset heart failure the HRs in stratified analysis. The HR were respectively in order of rising cTnT 1.28 (95 % CI 1.04–1.58 *p* = 0.02), 1.18 (95 % CI 0.94–1.48 *p* = 0.16) and 1.31 (95 % CI 1.03–1.67 *p* = 0.03). There was trend towards higher cTnT being associated with higher risk. However, the group with cTnT 70–200 ng/L was not statistically significant. To further examine this, we additionally re-grouped values into three groups by peak post cTnT and compared the group with cTnT ≤14 ng/L to a group with cTnT >14–70 ng/L (HR 1.1 (95 % CI 0.97–1.34 *p* = 0.11) and a group with cTnT above 70 ng/L (HR 0.92 (95 % CI 0.78–1.09 *p* = 0.35). When grouped in three groups there was no statistically significant differences.

**Fig. 5:**
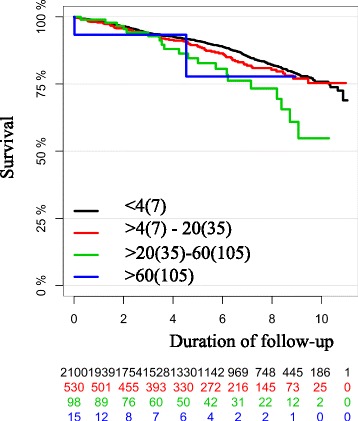
Kaplan-Meier curve of all-cause mortality by peak post procedural concentration of CK-MBmass. The values for women in μg/L are shown in the figure with values for men in parentheses. Duration of follow-up in years from index procedure. Below the Kaplan-Meier curve is shown the number of patients still available for follow-up in each group

There was no significant difference regarding all-cause mortality when analyzed in four groups after peak post-PCI CK-MBmass: <URL, between URL and 5 x URL (HR 1.96 (95 % CI 0.76–5.05 *p* = 0.16), between 5 and 15 x URL (HR 1.16 (95 % CI 0.55–2.44 *p* = 0.69) and above 15 x URL (HR 0.87 (95 % CI 0.55–1.37 *p* = 0.55) (Fig. 5). The results were unchanged in multivariable analyses that included age and gender. There was no clear pattern regarding the combined endpoint of all-cause mortality and new onset heart failure when analyzed in four groups after peak post-PCI CK-MBmass.: <URL, between URL and 5 x URL (HR 1.97 (95 % CI 1.19–3.27 *p* = 0.0088), between 5 and 15 x URL (HR 1.15 (95 % CI 0.77–1.72 *p* = 0.50) and above 15 x URL (HR 0.88 (95 % CI 0.68–1.15 *p* = 0.36). The results were unchanged in multivariable analyses that included age and gender.

## Discussion

The aim of this study was to examine the incidence of cardiac biomarker elevation according to the definition of peri-procedural MI given by the European Society of Cardiology-guidelines “Third Universal definition of MI” type 4a myocardial infarction, and to determine whether this carries independent prognostic value. The elevation of cTnT could be linked directly to the procedure, since only patients with normal baseline cTnT were included. Our population had an even distribution of patient characteristics with few differences and the differences in kidney function and previous MI do not seem clinically relevant since they do not have a clear pattern, and the differences are small. The only statistically significant and clinically meaningful differences between the groups were age and number of stents used. The incidence in our population using the cTnT cut off >5 x URL (cTnT >70 ng/L) was 15.2 %,

For the primary endpoint of all-cause mortality, we did not find that any interval of troponin was associated with an increased all-cause mortality. However, we did find a possible association between elevation of cTnT and the combined endpoint of new onset heart failure and all-cause mortality with the groups with cTnT >14–70 ng/L and >200 ng/L since these groups had borderline statistically significantly higher risk compared to the group with cTnT ≤14 ng/L. Regarding the combined endpoint of new onset heart failure and all-cause mortality and CKMB there was also a seemingly isolated risk associated with values between URL and 5x URL, but not with higher levels of CK-MB.

When using CK-MBmass the fraction of patients with elevated cardiac biomarkers was much lower than for cTnT (15.2 % versus 4.1 % respectively). The reason for this is probably, that cTnT is a more sensitive biomarker than CKMB mass. In contrast to some previous studies, we found that CK-MBmass was not an independent marker for prognosis [3, 4]. However, in our study we used CKMBmass, which is a more sensitive biomarker than CKMBcatalytic activity, and the >5 URL of CKMBmass corresponds to approximately an increase in CKMBcatalytic activity of 1–2 times URL; which in these studies did not show any prognostic information.

The fraction of elevated cardiac biomarkers found in our study is slightly higher than previously reported in meta-analysis by Testa et al. [12] They showed a detectable elevation of cTn after scheduled PCI in 28.7 % of 7578 patients (compared to 37.7 % in our study), and in 14.5 % of the patients cTn exceeded 3 x 99^th^ percentile URL (the previously used cut-off value) [13]. The data used by Testa et al. was of a more heterogeneous population with 7 studies including unstable angina and different assays including both cTnT and cTnI. Furthermore, different cut-off values for cTnT were allowed including 100 ng/L which even with 3 x URL (=300 ng/L) is far above the presently supported 70 ng/L. It seems likely, that these differences explain the lower occurrence of cTn above URL. In their meta-analysis there was a significant risk associated with elevation of cTn above 3 x URL, but this conclusion seems uncertain in our study of a more homogenous patient group. We found no association between biomarker elevation and all-cause mortality but a possible association between the combined endpoint of all-cause mortality and new onset heart failure. There is little doubt when examining the combined data that the presently used hsTnT is indeed highly sensitive and the present cut-off makes cTn elevation corresponding to peri-procedural MI a very common occurrence following elective PCI.

Peri-procedural MI can be difficult to rule out - as the symptoms, electrocardiographic changes, angiography and other imaging modalities can be uncertain due to older ischemic injuries and discomfort associated with the procedure itself. A study showed that on angiography only approximately 60 % of peri-procedural MI could be explained [14]. Clinicians must therefore rely considerably on cardiac biomarkers. However, this is not without difficulties, as a definite cut-off value for prognostic significance has not been established for the present sensitive cardiac biomarkers, and the nature of the association between elevated biomarkers after PCI and prognosis is still under discussion [2, 3, 15–21]. The question is whether increased all-cause mortality is caused by acute PCI-related myocardial necrosis or whether the elevations of biomarkers is due to diffuse cTn release functioning as a general indicator of increased risk. In a meta-analysis the relationship between elevated cTnI or cTnT above URL after elective PCI and all-cause mortality was shown to be statistically significant with increased long-term (3–67 months) all-cause mortality of 5.8 % compared to 4.4 % in the group without elevated cTn [22]. These studies were performed before the Third Universal definition of peri-procedural MI – type 4a – was defined. Furthermore, cut-offs of TnI or TnT were not given in the meta-analysis.

The issue is further opaqued by the diversity of PCI related causes of increased cTn such as re-perfusion of an already injured area, distal embolization, coronary dissection, coronary spasm, occlusion of a sidebranch or even major coronary artery, microvascular plugging, disruption of collateral flow, slow flow or no-reflow [23]. Some of these specific causes might be prognostically significant, but this might be overlooked, when all causes are examined as one general group with elevated biomarkers. In non-elective PCI the post-PCI elevation might be caused by a pre-PCI injury and an already rising baseline value as seen in acute coronary syndrome [15, 20, 21, 24]. It has been hypothesized that there are multiple risk factors. Marked elevations of biomarkers after elective PCI could be associated with large infarction area, which is known to be associated to increased risk of death by heart failure and malignant arrhythmias with worsening prognosis [25, 26]. On the contrary, smaller elevations might not represent an actually MI, but be an indicator of extensive coronary atherosclerosis and high procedural complexity, this is however, also known to be related to poorer long term outcome [18, 22, 27]. In previous studies, prognostic significance was demonstrated for post-PCI elevation of CK-MB [23, 28, 29]. However, cTn is now accepted as a more sensitive marker of myocyte necrosis than CK-MB [1, 18, 24] and therefore, it can be assumed that peri-procedural MI diagnosed on the basis of CK-MB had relatively larger probability of representing a severe MI than if based on cTn. However, in our study, there is no indication that CK-MBmass is a significant prognostic indicator, probably due to absolute low concentrations at >15 times URL in comparison to older studies, or superior to TnT.

To the best of our knowledge, the numerous studies previously describing elevated biomarkers after PCI all have considerably shorter follow up. Assuming that the prognostic significance of peri-procedural MI is due to myocardial scarring followed by development of heart failure and/or malignant arrhythmias, it seems reasonable that the difference in prognosis should progress over time. The shorter follow up of previous studies might lead to underestimation of risk, but based on our data this is a not the case.

### Limitations

Our study is a historic follow-up and is not able to report on any causality between peri-procedural MI and adverse events. Furthermore no data regarding symptoms, electrocardiographic changes or imaging is available to the study group so we cannot conclude whether the patients found with elevated cTnT ≥ 5 x URL also fulfilled the other defined criteria in the Third Universal Definition of Myocardial Infarction which includes patient reported symptoms, electrocardiographic or imaging suggestive of myocardial ischemia [1]. Due to the design of the study, it was necessary to use a surrogate marker for new-onset heart failure. Using hospitalization for heart failure as endpoint, was considered too insensitive, as a diagnosis of heart failure is known to be an unreliable marker with a sensitivity of about 25 % [30]. We expect most cases of overt heart failure to receive furosemide in Denmark, but acknowledge that some patients with obstructive lung disease and kidney disease may also receive this treatment, but it was deemed the best marker available when combined with hospital admittance due to heart failure.

It should be noted, that our data is from a strictly stable population and not “non-emergent” as used in many previous studies. Emergent is typically defined as procedures that have to be performed within hours of symptom onset. This group can include both recent non-ST-elevation myocardial infarction (NSTEMI) and unstable angina. NSTEMI can cloud the issue due to a rising baseline of cTn and also represents a higher risk of procedural complications. Therefore, the conclusions from this study should only be applied with great care to other than truly stable patients.

## Conclusion

The incidence of elevated biomarkers after elective PCI in patients with stable angina pectoris using the cut-off set by the Third Universal Definition of Myocardial Infarction (>5 x URL) was 15.2 % using cTnT and 4.1 % using CK-MBmass. The independent prognostic value of any cutoff for both cardiac biomarkers does not seem certain in neither short-term nor in long-term follow-up.

Our data suggest that routine measurement of cardiac biomarkers after elective PCI is not relevant unless procedural complications occur. We acknowledge that clinically driven measurement and observation after PCI in patients with stable angina pectoris is still relevant.

## Abbreviations

CCS, Canadian Cardiovascular Society classification of angina pectoris; CI, confidence interval; CK-MBmass, creatine kinase isoenzyme MB mass concentration; cTn, cardiac troponins; cTnT, troponin T; HR, hazard ratio; hsTnT, high sensitive troponin T; MI, myocardial infarction; NSTEMI, non-ST-segment elevation myocardial infarction; PCI, percutaneous coronary intervention; URL, upper reference limit.
